# *Strongyloides* Genetic Diversity among Humans, Dogs, and Nonhuman Primates, Central African Republic, 2016–2022

**DOI:** 10.3201/eid3203.250526

**Published:** 2026-03

**Authors:** Eva Nosková, Vladislav Ilík, Frédéric Stéphane Singa Niatou, Laurent Dumas, Terence Fuh, Jean-Francais Dicky, Terézia Kurucová, Vojtech Baláž, Klára Judita Petrželková, Barbora Pafčo

**Affiliations:** Department of Botany and Zoology, Masaryk University, Brno, Czech Republic (E. Nosková, V. Ilík, B. Pafčo); Masaryk University, Central European Institute of Technology, Brno (T. Kurucová); Czech Academy of Sciences, Institute of Vertebrate Biology, Brno (E. Nosková, V. Ilík, K.J. Petrželková, B. Pafčo); Czech Academy of Sciences, Biology Center, Institute of Parasitology, Ceske Budejovice, Czech Republic (K.J. Petrželková); WWF Central African Republic Programme Office, Dzanga Sangha Protected Areas, Bangui, Central African Republic (F.S. Singa Niatou, T. Fuh); Toulouse National School of Veterinarians, Toulouse, France (L. Dumas); University of Veterinary Sciences, Brno (V. Baláž)

**Keywords:** *Strongyloides*, parasites, zoonoses, One Health, primates, dogs, metabarcoding, Central African Republic

## Abstract

*Strongyloides stercoralis* nematode infection occurs in ≈600 million persons worldwide and is listed by the World Health Organization as a neglected tropical disease. Understanding zoonotic potential is critical, especially in areas where humans, domestic animals, and wildlife interact. We explored cross-species sharing of *Strongyloides* nematodes by analyzing fecal samples from humans, dogs, and nonhuman primates in the Dzanga-Sangha Protected Areas, Central African Republic. We detected positive samples by quantitative PCR and assessed genetic diversity through amplification of the 18S rRNA HVR-IV region and *cox1*, followed by high-throughput sequencing. *Strongyloides* prevalence was high in humans, dogs, and gorillas. *S. stercoralis* haplotype A nematode dominated in humans but appeared in dogs and apes, whereas *S. fuelleborni* nematode was present in all hosts. Shared species and haplotypes indicated zoonotic transmission. Our findings highlight the need for molecular surveillance and emphasize the role of dogs and nonhuman primates as reservoirs, complicating efforts to control infections in human populations.

Strongyloidiasis, caused by *Strongyloides* nematodes, is a zoonotic disease with public health and veterinary implications worldwide (*1*). The World Health Organization classifies strongyloidiasis among neglected tropical diseases requiring urgent control in endemic regions (*2*). Current estimates suggest that >600 million persons are infected globally, predominantly in tropical and subtropical areas (*3*). *Strongyloides stercoralis* and *S. fuelleborni* nematodes are the main species infecting humans; transmission typically occurs through transcutaneous exposure. More severe infections are primarily attributed to *S. stercoralis* nematodes, which is capable of autoinfection and can lead to severe systemic disease, particularly in immunocompromised persons; infection can result in death in extreme cases. Uncomplicated infections often manifest in gastrointestinal, pulmonary, or dermatological symptoms (*4*).

Molecular analyses of *S. stercoralis* nematodes have revealed 2 primary lineages: the potentially zoonotic lineage A, which involves dogs as potential reservoirs for human infection, and lineage B, which is largely restricted to canine hosts (*5*,*6*). Conversely, *S. fuelleborni* nematodes, although less common, are confined to nonhuman primates (NHPs) in Africa and Asia, occasionally spilling over to humans (*1*). Despite numerous documented cases, the true global prevalence of strongyloidiasis remains uncertain because of limited surveillance and underdiagnosis, compounded by the asymptomatic nature of many infections and the lack of standardized diagnostic tools (*7*,*8*).

The Central African Republic (CAR), one of the world’s most resource-constrained countries (*9*), ranks 191st out of 193 on the 2022 United Nations Human Development Index (*10*). Its tropical ecosystems, which are rich in biodiversity and wildlife (*11*), create favorable conditions for zoonotic disease circulation (*12*). Frequent interactions among humans, NHPs, domestic animals, and wildlife encourage the spread of infectious diseases (*13*), including *Strongyloides* nematodes (*14*). Dogs often act as ecologic bridges as a result of their hunting and scavenging behaviors (*15*). Soil-transmitted helminth infections remain among the most prevalent public health challenges in CAR (*16*); *Strongyloides* nematodes pose a particular concern for both human and NHP populations (*14*,*17*).

This study investigates the role of potential animal reservoirs in human *Strongyloides* infections within the Dzanga-Sangha Protected Areas (DSPA), CAR, where humans, NHPs, and domestic dogs interact closely at the human–wildlife interface. By using molecular genotyping, we analyzed the diversity and sharing of *Strongyloides* haplotypes among humans, NHPs, and dogs to inform control strategies in complex multihost systems.

## Materials and Methods

### Study Design and Participants

**Figure 1:**
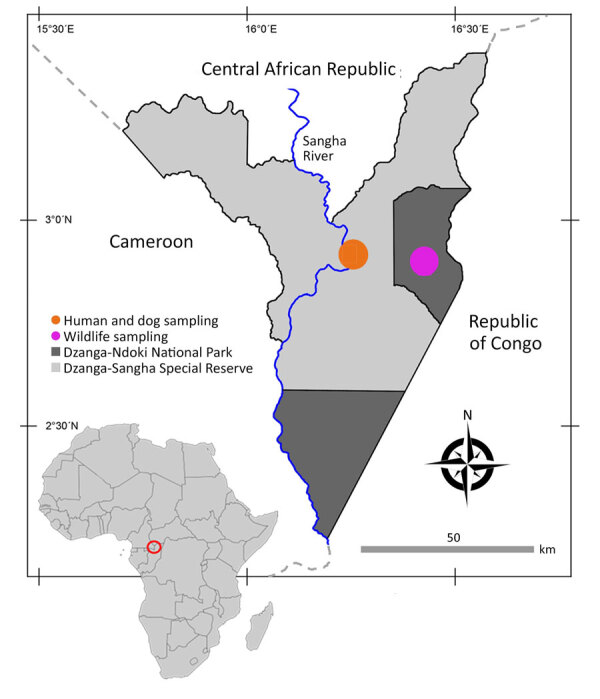
Location of study site in analysis of *Strongyloides* genetic diversity among humans, dogs, and nonhuman primates, Dzanga-Sangha Protected Areas, Central African Republic, 2016–2022. Inset shows location of Central African Republic in Africa. Figure adapted and adjusted from Hasegawa et al. (*18*).

The study was conducted in the DSPA in CAR (Figure 1) during 2016–2022, a location known for habituation of western lowland gorillas (*Gorilla gorilla gorilla*) to human presence and home of traditional BaAka hunter-gatherer communities living in close contact with wildlife. DSPA consists of several zones at varying levels of protection, including the strictly protected Dzanga-Ndoki National Park and the Dzanga-Sangha Special Reserve, a multiuse area where human activities are regulated to varying extents (Figure 1). The research complied with the legal requirements of the CAR and with all research, ethical, and sample transport approvals (Appendix 1).

Samples were collected from humans and domestic dogs from 2 villages located within the Special Reserve, whereas wildlife samples were collected in the National Park. Fresh stool samples were obtained from BaAka trackers who worked directly with NHPs in the National Park and also entered the Special Reserve (n = 18), as well as from humans residing in the villages (n = 32), who access the Special Reserve for daily activities such as gathering or hunting. Samples were also collected from dogs (n = 47), which were further categorized as hunting dogs entering the Special Reserve (n = 35) and guarding dogs (n = 12). Wildlife samples consisted of samples from western lowland gorillas (n = 101) at different levels of habituation, unhabituated central chimpanzees (*Pan troglodytes troglodytes*, n = 7), and a habituated group of agile mangabeys (*Cercocebus agilis*, n = 50). Samples were collected as part of health surveillance efforts (Appendix 2 Table 1). Approximately 1 g of feces was collected and immediately fixed in 96% ethanol and stored at –20°C before DNA isolation.

### Procedures

We dried fecal samples overnight at 37°C to evaporate the ethanol. We isolated total DNA using the PowerSoil DNA isolation kit (QIAGEN, https://www.qiagen.com). The extracted DNA was first screened by quantitative PCR (qPCR) targeting the small subunit (18S) rRNA gene specific to the genus *Strongyloides* (*19*) using the LightCycler 480 Real-Time PCR system (Roche, https://www.roche.com). We only included samples positive for *Strongyloides* in high-throughput library preparation. We used DNA extractions from *Strongyloides* PCR-negative feces and water as negative controls, whereas *S*. *stercoralis* first-stage larva was the positive control. All samples were prepared in technical PCR replicates (*20*). We prepared the high-throughput sequencing library using a 3-step PCR approach (Appendix 2 Table 2). We designed primers for the first PCR step, nested for the hypervariable region IV (HVR-IV) of the 18S rRNA gene and seminested for a portion of the mitochondrial cytochrome c oxidase subunit 1 gene (*cox1*), to increase sensitivity and ensure sufficient yield of amplicons for downstream sequencing (Appendix 1). In the second PCR step, we amplified the HVR-IV-18S rRNA and *cox1* (*21*). In the final PCR step, we applied Nextera primers with unique barcodes for each technical PCR replicate. We performed paired-end sequencing (2 × 300 bp) on the MGI DNBSEQ-G400 platform (MGI Tech, https://mgi-tech.eu).

We first demultiplexed raw fastq sequences and trimmed primer sequences using skewer version 0.2.2 (*22*). We then filtered, dereplicated, and denoised trimmed sequences and merged paired reads in R version 4.2.2 using the dada2 package (*23*). After processing, the final amplicon lengths were 255 bp for the 18S rRNA region and 217 bp for the *cox1*. During merging, we marked amplicon sequencing variants (ASVs) inconsistently present in both PCR technical replicates as potential artifacts and removed them from downstream analyses. We searched for corresponding sequences against the National Center for Biotechnology Information Nucleotide database (downloaded in February 2024) and excluded environmental, uncultured, <85% identity, and <90% coverage hits. We downloaded taxonomy using taxonomizer and used the created reference database to assign a taxonomic classification in our dataset through dada2’s Assign Taxonomy method (*24*).

### Statistical Analysis

We analyzed all data using the statistical software RStudio (https://www.rstudio.com). We assessed statistical differences in *Strongyloides* occurrence between trackers and villagers and between hunting and guard dogs using the χ^2^ test. We generated a bar plot to depict the proportion of *Strongyloides* haplotypes of HVR-IV-18S rRNA in each sample for better resolution. For *cox1* haplotypes, we constructed a median-joining network and visualized using Population Analysis with Reticulate Trees (PopART, https://popart.maths.otago.ac.nz). In addition, we performed a phylogenetic analysis using the MrBayes plugin in Geneious 9.1.5 (https://www.geneious.com) to complement the network approach and assess the phylogenetic placement of the detected haplotypes. To examine differences in α diversity, assessed as ASVs richness (number of ASVs per sample), we applied a generalized linear model (GLM) with a quasipoisson error distribution. We conducted posthoc pairwise comparisons using the Tukey test to identify specific differences among the studied host groups. We evaluated community composition diversity by analyzing the relative representation of HVR-IV-18S rRNA and *cox1* ASVs using Bray-Curtis ecologic distances. We visualized the clustering patterns with principal coordinate analysis (PCoA). To test for interspecific differences in *Strongyloides* nematode community composition among hosts, we performed a permutational analysis of variance (PERMANOVA), followed by similarity analysis (ANOSIM).

## Results

### *Strongyloides* qPCR Detection

The number of *Strongyloides*-positive samples by qPCR was high across all studied host species; rates were 76% (38/50) in humans, 60% (28/47) in dogs, 59% (58/101) in gorillas, 43% (3/7) in chimpanzees, and 38% (19/50) in mangabeys. Among humans, BaAka gorilla trackers exhibited a slightly higher, although nonsignificant (χ^2^ = 0.83; p = 0.36), prevalence of infection (83.3% [15/18]) than that observed in villagers (71.9% [23/32]). Similarly, hunting dogs, which actively venture into the forest, showed a significantly (χ^2^ = 12.32; p = 0.0004) higher prevalence (74.3% [26/35]) than did guard dogs, which remain in villages (16.7% [2/12]). All qPCR-positive samples were further successfully sequenced with both targets (HVR-IV-18S rRNA and *cox1*), except for 3 mangabey samples that were near the detection threshold (cycle threshold ≈33–34), which likely explains their sequencing failure.

### *Strongyloides* HVR-IV-18S rRNA Diversity

Genotyping identified a total of 11,472,181 high-quality HVR-IV-18S rRNA reads, with a median sequencing depth of 74,200 (range 1,221–302,518) per sample. Taxonomic analysis identified a total of 24 ASVs (Table). Six clustered into already known *Strongyloides* haplotypes: haplotype A of *S. stercoralis* and haplotypes K, L, M, P, and T of *S. fuelleborni*. Five *S. fuelleborni* ASVs did not match with any previously described haplotypes. Those ASVs were detected in only 9 samples (9/140 [6.4%]). Three haplotypes were host-specific (detected exclusively in human, dog, or gorilla), whereas 2 haplotypes were shared (1 between gorilla and mangabey and the other between human and dog). Six ASVs were classified as uncharacterized *Strongyloides* species and were found in 14 samples (14/140 [10%]). Three ASVs were detected in a single host, each occurring exclusively in mangabeys, whereas the remaining 3 were shared (2 between humans and dogs, and 1 within mangabeys). Last, 7 unassigned ASVs, detected in 19% of the samples, were tentatively classified as being most closely related to the order Rhabditida.

**Table:** Prevalence of individual *Strongyloides* hypervariable region IV 18S rRNA haplotypes assessed from quantitative PCR–positive samples across host species in study of *Strongyloides* genetic diversity, Central African Republic, 2016–2022

Haplotype	No. (%)				
	Humans, n = 38	Dogs, n = 28	Gorillas, n = 58	Chimpanzees, n = 3	Mangabeys, n = 16
*S. stercoralis* A	28 (73.7)	7 (25)	7 (12.1)	0	0
*S. fuelleborni* K	9 (23.7)	6 (21.4)	4 (6.9)	0	0
*S. fuelleborni* L	38 (100)	26 (92.8)	54 (93.1)	3 (100)	13 (81.3)
*S. fuelleborni* M	7 (18.4)	9 (32.1)	0	0	0
*S. fuelleborni* P	1 (2.6)	1 (3.6)	38 (65.5)	3 (100)	10 (62.5)
*S. fuelleborni* T	1 (2.6)	0	0	0	0
*S. fuelleborni* unclassified	2 (5.3)	3 (10.7)	2 (3.4)	0	2 (12.5)
*Strongyloides* unclassified	3 (7.9)	4 (14.3)	3 (5.2)	0	4 (25)

**Figure 2:**
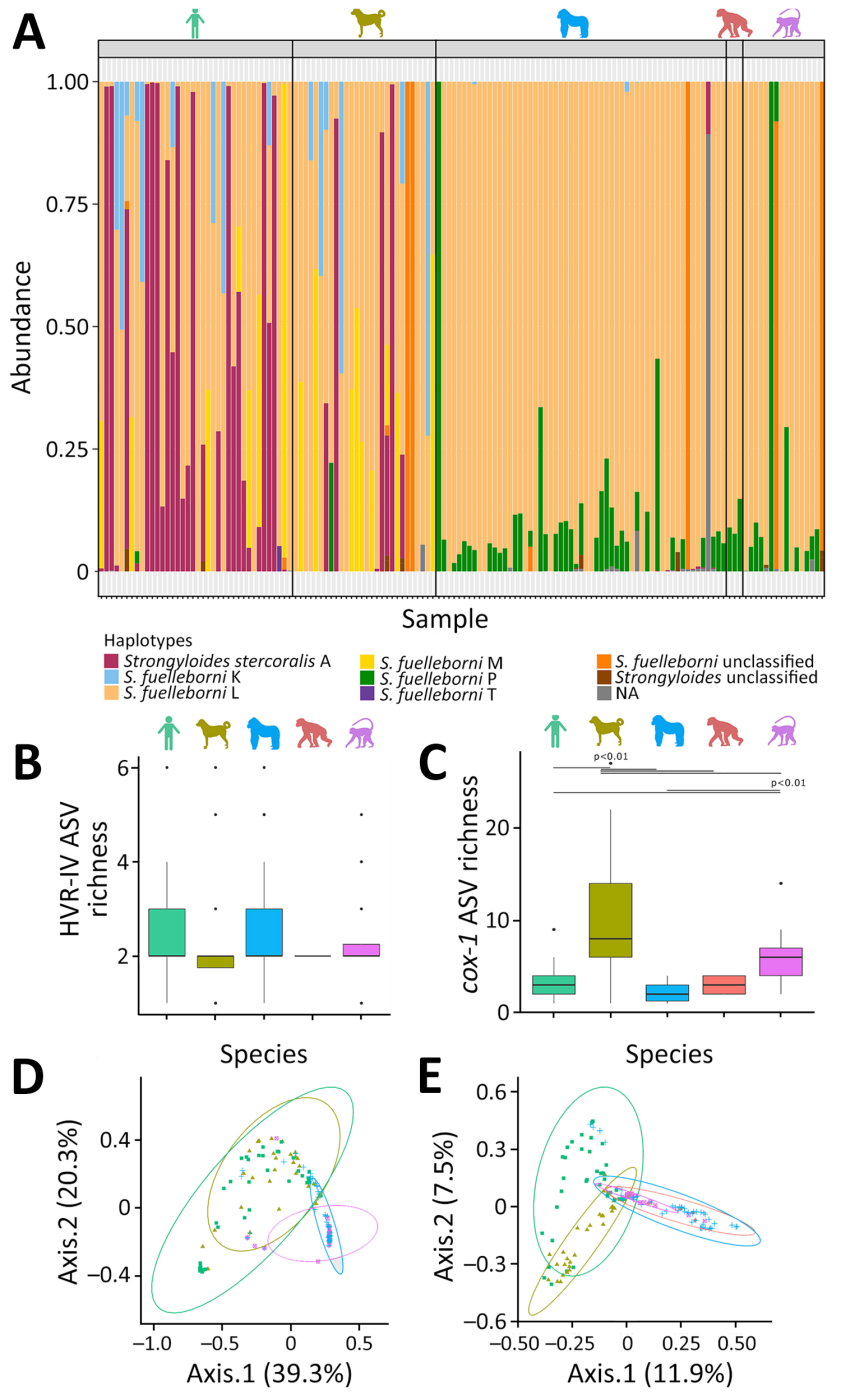
Relative community composition of *Strongyloides* HVR-IV-18S rRNA haplotypes across examined hosts in study of *Strongyloides* genetic diversity among humans, dogs, and nonhuman primates, Dzanga-Sangha Protected Areas, Central African Republic, 2016–2022. A) Relative abundance of haplotypes shown as color panels; each column represents a single sample. B) Boxplot showing the α diversity of *Strongyloides* HVR-IV 18S rRNA haplotypes, represented by the number of ASVs per sample (dots) grouped by host species. C) Boxplot showing the α diversity of *Strongyloides cox1* haplotypes represented by the number of ASVs per sample (dots) grouped by host species. D) Beta diversity of *Strongyloides* HVR-IV 18S rRNA haplotype communities, based on Bray-Curtis ecologic distances (relative abundance of reads), visualized using principal coordinate analysis ordination diagrams. E) Principal coordinate analysis showing the β diversity of *Strongyloides cox1* haplotypes. Color silhouettes (A-C) and data points (D, E) indicate host species: light green, human; olive green, dog; blue, gorilla; red, chimpanzee; violet, mangabey. ASV, amplicon sequencing variant; HVR-IV, hypervariable region IV.

Overall, *S. fuelleborni* nematodes dominated across all studied hosts; haplotype L was the most prevalent variant (93.8% of total prevalence). Potentially zoonotic haplotype A of *S. stercoralis* was detected in 29% of samples from humans, dogs, and gorillas (Table). Relative abundances bar plot of *Strongyloides* HVR-IV-18S rRNA ASVs shows interspecific differences in *Strongyloides* community composition depending on host species (Figure 2). Humans were predominantly infected with *S. fuelleborni* haplotype L and *S. stercoralis* haplotype A (Table; Figure 2, panel A). *S. stercoralis* haplotype A was detected less frequently in BaAka gorilla trackers (67%) than in their village-dwelling relatives (78%), although that difference was not statistically significant (χ^2^ = 0.63; p = 0.43); haplotype A was absent in guard dogs but present in 30% of hunting dogs. NHPs were mainly infected with *S. fuelleborni* haplotype L. Unclassified *S. fuelleborni* and other unassigned *Strongyloides* variants were predominantly found in mangabeys (Table).

### Zoonotic Potential of *Strongyloides* Nematode on the Basis of HVR-IV-18S rRNA

The highest number of *Strongyloides* HVR-IV-18S rRNA ASVs (8) was shared between humans and dogs, accounting for 33.3% of all observed ASVs. Those included haplotype A of *S. stercoralis*, haplotypes K, L, M, and P of *S. fuelleborni*, 1 unclassified *S. fuelleborni* (ASV_19), and 2 unidentified *Strongyloides* spp. (ASV_14 and ASV_21). Four ASVs (16.6% of all observed ASVs), haplotype A of *S. stercoralis,* and haplotypes K, L, and P of *S. fuelleborni* were shared between humans and NHPs. Haplotype A of *S. stercoralis* and haplotypes K, L, and P of *S. fuelleborni* were shared across humans, dogs, and gorillas (Figure 2, panel A).

### Differences in *Strongyloides* HVR-IV-18S rRNA Communities

The number of ASVs per sample did not differ significantly among host species, as determined by a GLM (p>0.1) and subsequent Tukey test (Figure 2, panel B). However, the PCoA diagram based on Bray-Curtis ecologic distances revealed distinct differences in *Strongyloides* community composition among humans, dogs, and NHPs (Figure 2, panel D). Those differences were further statistically confirmed by PERMANOVA (F_(4,137)_ = 11.372; p = 0.001) and ANOSIM (R = 0.3435; p = 0.001) tests. The overlap in *Strongyloides* communities was greater between humans and dogs, whereas communities within NHPs were more similar but distinct from those of humans and dogs (Figure 2, panel D).

### *Strongyloides cox1* Diversity and Zoonotic Potential

**Figure 3:**
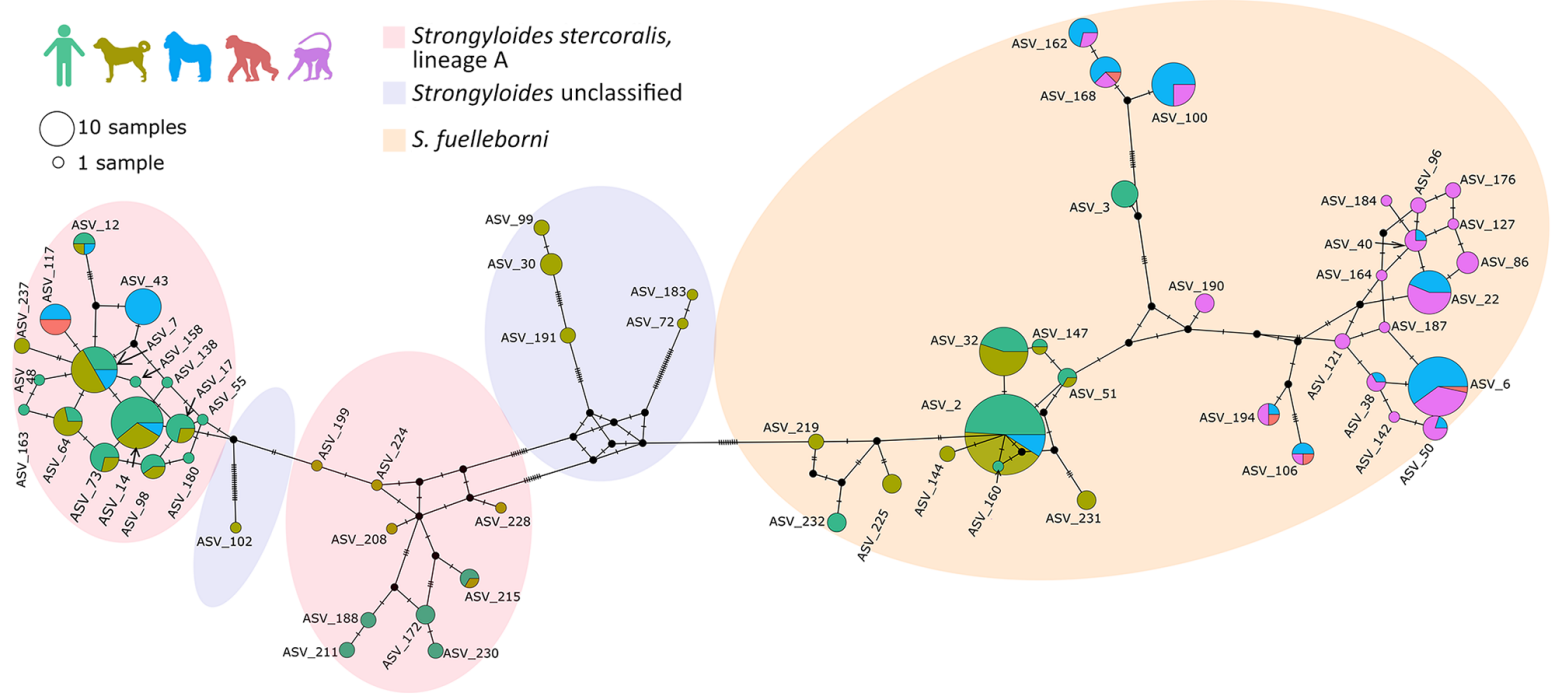
Median-joining *Strongyloides* haplotype network for the mitochondrial *cox1* gene studied in various host species in study of *Strongyloides* genetic diversity among humans, dogs, and nonhuman primates, Dzanga-Sangha Protected Areas, Central African Republic, 2016–2022. Each circle represents 1 haplotype; circle size indicates the number of hosts harboring the respective haplotype. The colors inside the circles indicate the host species: light green, human; olive green, dog; blue, gorilla; red, chimpanzee; violet, mangabey. The hatch marks beside the branches indicate the number of mutation steps between the haplotypes. Missing haplotypes are indicated by small black circles. Shaded ovals indicate species/lineage type. ASV, amplicon sequencing variant.

**Figure 4:**
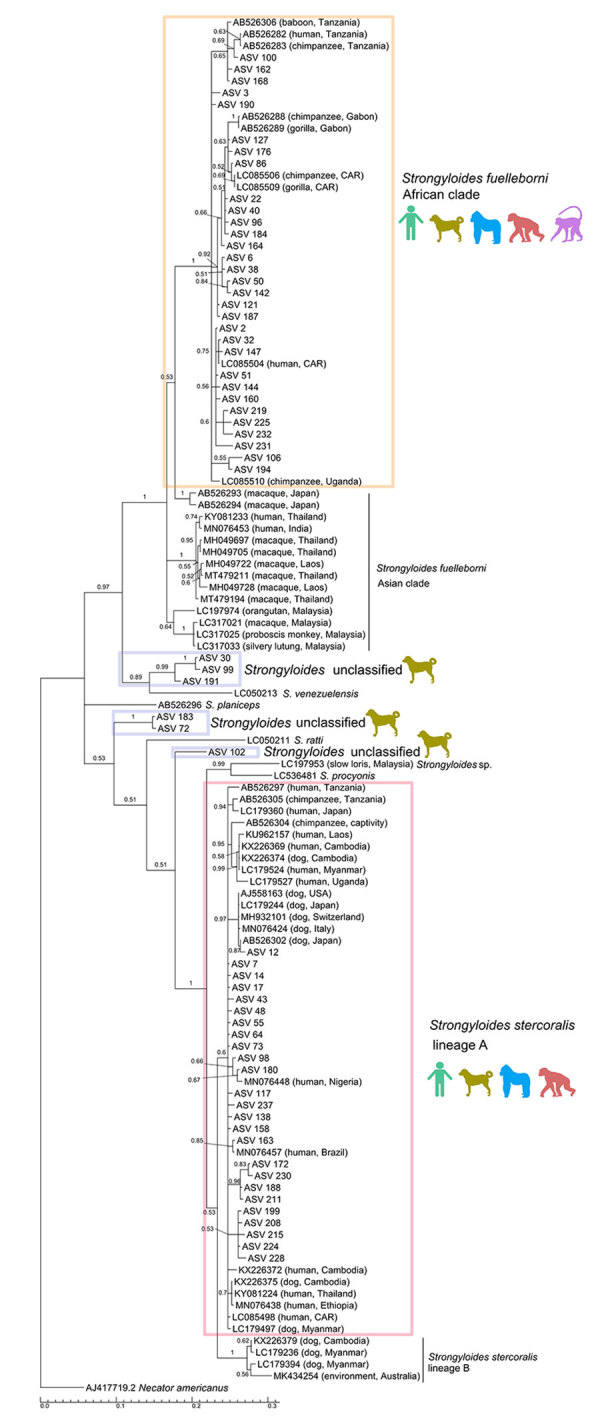
Bayesian phylogenetic tree based on *Strongyloides* spp. *cox1* (700 bp) sequences derived from study of *Strongyloides* genetic diversity among humans, dogs, and nonhuman primates, Dzanga-Sangha Protected Areas, Central African Republic, 2016–2022. Tree also includes sequences downloaded from GenBank; accession numbers are provided. The alignment was performed in Geneious (https://www.geneious.com) using the general time-reversible model of nucleotide substitution with gamma distribution. Branch lengths indicate expected substitutions per site. *Necator americanus* (accession no. AJ417719.2) was included as an outgroup. Node support was estimated from 10^6^ iterations. Color silhouettes indicate host species: light green, human; olive green, dog; blue, gorilla; red, chimpanzee; violet, mangabey. Colored boxes indicate species/lineage type. ASV, amplicon sequencing variant; CAR, Central African Republic.

We identified a total of 3,381,999 reads based on *cox1*; the median sequencing depth was 2,314 (range 75–56,108) per sample. Taxonomic assignment revealed 62 *Strongyloides* ASVs and 72 unassigned variants, which we tentatively classified as closest to the order Rhabditida. The 62 *Strongyloides* ASVs were used to construct a median-joining phylogenetic network (Figure 3) and Bayesian phylogeny (Figure 4).

The nucleotide diversity was high (N = 0.076), and 52 of the ASVs were parsimony informative. The haplotype network consisted of 25 *S. stercoralis*, 31 *S. fuelleborni*, and 6 unclassified *Strongyloides* ASVs. The network revealed divergence in ASVs on the basis of host specificity. *S. stercoralis* ASVs were primarily shared between humans and dogs, with occasional overlap with gorillas. Haplotype ASV_7 was most common in dogs, whereas ASV_14 was most frequent in humans. Chimpanzees shared only 1 haplotype (ASV_117) with gorillas, and intriguingly, haplotype ASV_43 was found exclusively in gorillas (Figure 3). In the phylogenetic tree, all ASVs of *S*. *stercoralis* cluster within potentially zoonotic lineage A (Figure 4). *S. fuelleborni* ASVs all clustered to *S. fuelleborni* Africa clade (Figure 4) and displayed host-specific divergence (Figure 3). Humans shared *S. fuelleborni* haplotypes mostly with dogs and occasionally with gorillas, whereas other haplotypes were restricted to NHPs. The ASV_2 haplotype was most common in both humans and dogs, whereas ASV_6 was predominant in gorillas and mangabeys. Haplotypes of unclassified *Strongyloides* species were detected only in dogs (Figure 4). The first cluster, consisting of ASV_30, ASV_99, and ASV_191, formed a well-supported branch closely related to *S. venezuelensis*. The second cluster, represented by ASV_183 and ASV_72, grouped between *S. planiceps* and *S. ratti* but with low nodal support. The final lineage, ASV_102, formed a separate, also weakly supported branch positioned between *S. ratti*, *S. procyonis*, and unclassified *Strongyloides* spp. found in Bornean slow lorises. Overall, the median-joining phylogenetic network and Bayesian phylogeny were highly complementary, revealing host-associated divergence in *S. stercoralis* and *S. fuelleborni* and highlighting distinct clustering of unclassified *Strongyloides* taxa.

### Differences in *Strongyloides* *cox1* Communities

*cox1* ASV richness differed significantly among the studied hosts (GLM F_(4,134)_ = 253.93; p<0.0001). Tukey posthoc testing revealed that dogs differed significantly from all other hosts (p<0.01 for all pairwise comparisons), whereas mangabeys also differed significantly from humans and gorillas (p<0.01) (Figure 2, panel C). We observed no significant differences between the remaining host pairs. The PCoA diagram based on Bray-Curtis ecologic distances further confirmed clear differences between humans, dogs, and NHPs (Figure 2, panel E), supporting the findings of the haplotype network analysis. Those differences in the composition of *Strongyloides* infections between host species were further statistically confirmed by PERMANOVA (F_(4,135)_ = 11.192; p = 0.001) and ANOSIM (R = 0.3438; p = 0.001) tests.

## Discussion

We investigated *Strongyloides* nematodes diversity and haplotype sharing among humans, dogs, and NHPs cohabiting in the DSPA, CAR, to assess zoonotic potential and multihost transmission (*25*). A previous study from the DSPA investigated *Strongyloides* nematodes in humans, gorillas, and chimpanzees, reporting *S. stercoralis* exclusively in humans and identifying different haplotypes of *S. fuelleborni* in gorillas and chimpanzees than those found in humans (*14*). However, that study was limited by a small sample size of larvae and did not include dogs, which are potential hosts. Our study highlights the critical importance of employing modern diagnostic approaches and examining a broader range of hosts from the same locality to better understand *Strongyloides* sharing in the ecosystem.

Our genotyping approach revealed a remarkable degree of genetic diversity within *Strongyloides* species, reflecting the complexity of their populations, and further identified the sharing of specific haplotypes across different host species. Of note, *S. fuelleborni*, likely originating from NHPs, dominated across all hosts; haplotype L was the most prevalent variant. Zoonotic *S. stercoralis* haplotype A was observed primarily in humans, dogs, and gorillas. This variation in prevalence underscores the host-specific transmission dynamics and the role of local ecologic factors in determining *Strongyloides* distribution. Those findings emphasize the need for a One Health approach (*26*) because of the zoonotic potential of *S. stercoralis* nematodes and the species’ strong connection to the environment.

Despite the identification of dominant haplotypes, the proportion of *Strongyloides* sequences remains unclassified, highlighting the limitations of short-marker–based phylogenetic inference. Unclassified *S. fuelleborni* haplotypes were detected in all host species except chimpanzees, for which only a limited dataset was available. Those sequences likely reflect previously undetected diversity, because additional haplotypes have been reported with expanded sampling. Given the limited data currently available from sites in Africa (*27*), further haplotypes are expected to emerge as sampling across hosts and regions improves.

Several unclassified ASVs clustered with *S. venezuelensis* with high nodal support, whereas other unclassified ASVs formed clusters with very low support, rendering their classification unreliable. The placement of such lineages, based solely on short *cox1* fragments, is highly challenging, because those sequences might lack sufficient phylogenetically informative sites to resolve relationships among closely related or cryptic taxa. Similar issues have been reported previously (e.g., short *cox1* fragments were insufficient for precise taxonomic resolution of cryptic *Strongyloides* in dogs [*28*]). Therefore, integrating comprehensive genetic data, such as longer mitochondrial sequences or genomic information from individual larvae, with morphological analyses of larvae and adult or paratenic females is crucial for resolving the taxonomy and evolutionary relationships of unclassified *Strongyloides* spp.

We recognize the inherent limitations of single-locus genotyping, because reliance on a single marker provides a restricted representation of genetic diversity and might not reflect genome-wide variation. That limitation means substantially larger host sample sizes are required to achieve the same statistical power and robustness that whole-genome data can provide, because only by averaging across many individuals can stochastical effects at a single locus be mitigated. We also acknowledge that our reliance on fecal DNA, although advantageous as a noninvasive sampling method, can introduce issues related to DNA degradation, contamination, and allelic dropout, further constraining data quality. Thus, our conclusions should be interpreted with caution and viewed as complementary to, rather than a substitute for, genome-wide analyses.

*Strongyloides* communities were more similar between humans and dogs than between either of those hosts and NHPs. The highest proportion of shared haplotypes was observed between humans and dogs, accounting for 33.3% of all detected ASVs. This pattern supports the involvement of dogs in the circulation of *S. stercoralis* nematodes alongside humans in the DSPA (*5*,*6*). Consistent with this pattern, *S. stercoralis* haplotype A predominated in humans, suggesting that human-associated circulation represents a key component of *Strongyloides* nematodes occurrence in this setting and that dogs potentially act as secondary hosts.

Unexpectedly, sharing between humans and dogs was also observed for *S. fuelleborni* haplotypes K and M, a species previously considered to circulate primarily in NHPs (*14*). The presence of haplotype M in both humans and dogs, with higher prevalence in dogs, is notable given its limited geographic reporting to date in humans, restricted to Senegal (*21*) and CAR (*14*). The high prevalence in dogs suggests a potential contribution to the environmental dissemination of *S. fuelleborni* nematodes within DSPA, although spurious infections associated with coprophagy cannot be excluded (*29*). Experimental infections of dogs with *S. fuelleborni* nematodes have been demonstrated (*30*), but natural infections remain unconfirmed, and fecal metabarcoding alone cannot resolve the definitive host status of dogs.

ASVs shared between humans and NHPs included *S. stercoralis* haplotype A and *S. fuelleborni* haplotypes K, L, and P, accounting for 16.6% of all observed ASVs; all of those were also detected in dogs. *Strongyloides fuelleborni* haplotype L, which is well established across all studied hosts, was previously reported only in gorillas (*14*) and in African vervets on St. Kitts (*31*). Although its origin remains speculative, its presence across host species suggests extensive host sharing within the DSPA. In contrast, haplotype P was predominantly detected in NHPs, which likely represent a key source of exposure for humans and dogs. Together, these patterns indicate shared circulation of *Strongyloides* nematodes among humans, dogs, and NHPs in the DSPA.

Given the soil-transmitted nature of *Strongyloides* nematodes and the close coexistence of hosts in DSPA, environmental exposure represents a major challenge for disease control (*4*). Although chemotherapy is effective in the short term to treat strongyloidiasis (*32*), high reinfection rates, autoinfection, free-living stages in soil, multiple host species, and emerging concerns about drug resistance complicate long-term elimination, particularly in tropical settings (*33*,*34*), highlighting the need for a comprehensive strategy. A crucial aspect of controlling strongyloidiasis is recognizing the role of wildlife in transmission. Because treating wildlife such as free-ranging NHPs is not feasible, effective control requires a One Health approach that considers humans, domestic animals, and the environment (*26*). Practical measures, including proper latrine use and use of footwear to reduce soil contact, should complement treatment of infected persons (*35*). Ivermectin remains the treatment of choice (*33*), but its use must be carefully considered in regions with high *Loa loa* microfilaremia prevalence (*36*), as in CAR. Overall, the potential for rapid reinfection from multiple hosts underscores the need for integrated control strategies.

In conclusion, the close coexistence of multiple host species sharing *Strongyloides* infections in the DSPA likely contributes to the persistence of the parasite in this environment. Expanding investigations beyond humans to include domestic animals and wildlife is therefore essential, and our findings highlight the relevance of a One Health approach that integrates those host groups (*37*). The observed overlap of *S. stercoralis* and *S. fuelleborni* haplotypes among humans, dogs, and NHPs reflects the complexity of host associations within this ecosystem. By adopting a multihost diagnostic approach, we provide direct insights into *Strongyloides* nematodes sharing among hosts in the DSPA, informing more realistic and sustainable control strategies.

Appendix 1 Additional information about *Strongyloides* genetic diversity among humans, dogs, and nonhuman primates, Central African Republic, 2016–2022

Appendix 2 Additional information used in study of *Strongyloides* genetic diversity among humans, dogs, and nonhuman primates, Central African Republic, 2016–2022
