# Supplementary material for: Strongyloides Genetic Diversity among Humans, Dogs, and Nonhuman Primates, Central African Republic, 2016–2022
Source: Emerg Infect Dis. 2026 Mar;32(3):349–59. doi: 10.3201/eid3203.250526 (PMC13016005; doi:10.3201/eid3203.250526)
Supplement: Appendix 1 — Additional information about Strongyloides genetic diversity among humans, dogs, and nonhuman primates, Central African Republic, 2016–2022 [file 25-0526-Techapp-s1.pdf]

Article DOI: https://doi.org/10.3201/eid3203.250526

EID cannot ensure accessibility for supplementary materials supplied by authors. Readers who have difficulty accessing supplementary content should contact the authors for assistance.

Strongyloides Genetic Diversity among Humans, Dogs, and Nonhuman Primates, Central African Republic, 2016–2022

Appendix 1

Sample approvals

Samples were collected non-invasively and in compliance with DSPA research protocols and site regulations. All work was approved according to the rules and regulations from the Ministère de l'Enseignement Supérieur, de la Recherche Scientifique et de l'Innovation Technologique' (Central African Republic). The collection of human samples was also approved by Ethical Commission of the Biology Centre of the Academy of Sciences, Ceske Budejovice, Czech Republic. All human participants were volunteers who were informed about soil-transmitted nematodes, the study's objectives, proper methods for collecting uncontaminated samples and provided consent for analyses of their samples. As the sampling aimed to assess parasite loads, the volunteers were provided with subsequent treatment by local healthcare institutions. The transport of samples from dogs and non-human primates was approved by the State Veterinary Authority of the Czech Republic.

Development of the primers used for the first-step PCR

Primers for the first-step PCR (nested for HVRIV-18S rRNA and semi-nested for *cox1*) were designed using Geneious 9.1.5 (www.geneious.com) and validated using Primer-BLAST (https://www.ncbi.nlm.nih.gov/tools/primer-blast/). The primers were designed based on sequences from multiple *Strongyloides* species to maximize coverage across the genus. Specifically, sequences from 16 taxa were used: *S. stercoralis*, *S. fuelleborni*, *S. procyonis*, *S. cebus*, *S. ransomi*, *S. callosciuresus*, *S. robustus*, *S. mirzai*, *S. myopotai*, *S. akbari*, *S.*

papillosus, *S. vituli*, *S. ophidiae*, *S. westeri*, *S. suis*, and *S. planiceps*. The designed primers amplified a 758 bp fragment of the 18S rRNA gene and a 253 bp fragment of the *cox1* gene.
